# Novel Gene Mutations Causing Primary Congenital Glaucoma

**Published:** 2010-04

**Authors:** Sepehr Feizi

**Affiliations:** Ophthalmic Research Center, Labbafinejad Medical Center, Shahid Beheshti University of Medical Sciences, Tehran, Iran

Primary congenital glaucoma (PCG) is characterized by an anatomical defect in the trabecular meshwork (trabeculodysgenesis) and onset in the neonatal or infantile period, generally before the age of 3 years. The developmental anomaly in the anterior chamber angle manifests by increased intraocular pressure, corneal edema, excessive tearing, photophobia, enlargement of the globe (buphthalmos) and corneal opacification. It is the most severe form of glaucoma, having both sporadic and familial patterns with an incidence of 1:10,000 to 1:2,500 of live births. In familial cases, the pattern of inheritance is usually autosomal recessive.

So far, three PCG loci have been identified by linkage analysis in multiple affected families: GLC3A, GLC3B, and GLC3C. The gene associated with GLC3A, cytochrome P450 1B1 (*CYP1B1*), is a member of the cytochrome P450 superfamily of genes and was the first gene linked to PCG in 1998. Its mutations are responsible for the disease in 20–100% of patients from different ethnicities. The rate of *CYP1B1* mutations in Iranian PCG subjects has been reported to be 70%. Although physiological studies have confirmed that mutations in *CYP1B1* can cause the disease, the pathway by which it affects normal development of the anterior chamber remains unknown. Additionally, mutations in myocilin (*MYOC*), a gene generally associated with early onset (juvenile) primary open angle glaucoma, have occasionally been reported in PCG.

In late 2009, the second gene for PCG was reported by two research groups, one of which was Iranian. Narooie-Nejad and colleagues recently performed autozygosity mapping in three Iranian PCG families and encountered two novel mutations in a gene overlapping with the GLC3C locus on chromosome 14q24.2–14q24.3. They reported two disease-segregating loss of function mutations in *LTBP2*, namely p.Ser472fsX3 and p.Tyr1793fsX55. The latter mutation causes a defect close to the C-terminal of the encoded protein. Ali et al independently reported null mutations in *LTBP2* in PCG patients. The gene itself encodes latent transforming growth factor beta binding protein 2 (LTBP2) which is expressed in human eyes within the trabecular meshwork and ciliary processes. Abnormal expression of *LTBP2* is thought to be relevant to the etiology of PCG.

*LTBP2* contains 36 exons and encodes a matrix protein containing 1,821 amino acids. This protein is a member of a superfamily composed of fibrillins and LTBP proteins. These proteins contain tandem arrays of epidermal growth factor-like motifs, interspersed with characteristic transforming growth factor beta (TGF-β) binding protein-like motifs. TGF-βs constitute a family of potent multifunctional cytokines that modulate many cellular and physiologic processes. They exist extracellularly as latent complexes awaiting to be activated by appropriate signals. Although the precise function of LTBP2 remains unknown, there is evidence for this protein to have roles in tissue repair processes and cell adhesion. Mutations in the related gene can affect the structure and function of its protein and interfere with binding to fibrillin 1 and fibulin 5, leading to changes in the elastic properties of the ciliary processes and the trabecular meshwork. These alterations may directly or indirectly affect aqueous fluid outflow contributing to the PCG phenotype.
